
JOURNAL INFORMATION
==============================
NLM Title Abbreviation: J Can Health Libr Assoc
Journal ID: J Can Health Libr Assoc
NLM Journal ID: 101229936
Journal ID: JCHLA

The Journal of the Canadian Health Libraries Association

EISSN: 1708-6892
Publisher: Canadian Health Libraries Association / Association des bibliotèques de la santé du Canada

ARTICLE INFORMATION
==============================
PMCID: PMC12352450
DOI: 10.29173/jchla29892
Article ID: JCHLA-46-078
Article version: 1
Subjects: Abstracts

CHLA 2025 CONFERENCE POSTERS / ABSC CONGRÈS 2025 AFFICHES

Electronic publication date: 2025 Aug 1
Publication date: 2025 Aug
Volume: 46
Issue: 2
First page: 78
Last page: 83
Copyright: © Fournier
Copyright year: 2025
License: This article is distributed under a Creative Commons Attribution License: https://creativecommons.org/licenses/by/4.0/
License URL: https://creativecommons.org/licenses/by/4.0/

==============================

PP = Poster Presentation

## PP1. A fork in the road: machine learning classifier or methodological search filter to identify systematic reviews?

Melissa Severn, Alissa Epworth, Romney Adams
DistillerSR

**Background:** Systematic reviews (SRs) can be retrieved in several ways. One approach is a search filter designed to retrieve SRs in bibliographic databases such as PubMed. Another utilizes machine learning (ML) to sort articles into two mutually exclusive classes in online platforms such as DistillerSR. **Objective:** To test the performance of a binary ML classifier designed to identify SRs in DistillerSR against search filters designed to retrieve SRs in PubMed. **Methods:** Umbrella reviews will be identified, included SRs will be extracted to create a reference set. Each SR in the reference set will be verified as indexed in PubMed. Two SR search filters will be tested: a narrow SR filter and a broad SR filter. A binary ML classifier developed by DistillerSR to identify SRs will be used, informed by the reference set and a seed of non SR articles. The number of SRs from the reference set retrieved by the two search filters, and the number of SRs from the reference set correctly identified as a SR by the ML classifier will each be recorded discretely. **Results:** Relative recall, precision and F1 score will be calculated for each set. Recommendations will be made on when to apply a search filter or ML classifier to a search. **Conclusions:** Different approaches to limiting search results to specific study designs can influence the overall search strategy. The objectives of the project and resource requirements need to be considered when deciding on the approach.

## PP2. A review of library services and supports for the University of Prince Edward Island's (UPEI) growing One Health programs

**Introduction:** One Health, the study of the interconnectedness of human and animal health with environmental health, is a growing discipline of study at many universities [1]. With the establishment of a Faculty of Medicine, UPEI now hosts programs in all areas of One Health, including the School of Climate Change and Adaptation and the Atlantic Veterinary College. Limited literature exists on library supports specific to One Health programs. **Description:** After UPEI established a Faculty of Medicine and signed a Planetary Health (aka One Health) declaration, the Robertson Library reviewed its services and programs to ensure sufficient support for the discipline. Staffing, collections, and information literacy (IL) instruction were identified as key areas to augment. **Outcomes:** Technician staffing was adequate, however, a librarian position was restructured to include liaising with the veterinary medicine program alongside environmental disciplines, in addition to a new medical librarian position. Health resources were expanded through the medical program funding, and subject-specific funds were used for increasing One Health collections. New resources were identified, and proposals were brought forward. IL instruction was varied across the programs, but heavily present in veterinary medicine. **Discussion:** As a small institution, supporting the discipline of One Health requires collaboration. While IL occurs in One Health disciplines, there is room to increase instruction in climate change and environmental studies.

Future plans include continued efforts in IL for the first cohort of the medical program (to begin fall 2025) as well as the creation of a One Health subject guide.

**References:**
1. World Health Organization. One Health [Internet]. 2017 [cited 2025 Jan 15]. Available from: https://www.who.int/news-room/questions-and-answers/item/one-health.

## PP3. Building a healthier Alaska: connecting Alaskans to essential resources through the Alaska Medical Library

Anna Bjartmarsdottir, Jennifer McKay
University of Alaska

**Overview:** The Alaska Medical Library (AML), the state's only medical library, plays a critical role in providing reliable health information to diverse populations across Alaska, particularly in remote communities with limited access to internet or travel options. Located at the University of Alaska Anchorage (UAA), AML serves both academic and healthcare communities statewide, helping combat health misinformation and improve health literacy. AML's initiatives include a variety of outreach programs, partnerships, and resource development efforts aimed at increasing access to trustworthy health information. **Collaborative projects:** In collaboration with UAA's Area Health Education Center (AHEC), AML delivers health information to healthcare professionals, enhancing its visibility in Alaska's healthcare landscape. AML has also worked with the Peer Leader Navigators (PLN) program to produce multilingual health literacy materials, such as a vaccine confidence coloring book, which were distributed statewide via the Statewide Library Electronic Doorway (SLED). Additionally, AML's involvement in a health misinformation project led to the creation of a LibGuide with resources on combating misinformation, which was made available to Alaskans through SLED. **Ongoing work:** Despite the conclusion of several grant-funded projects, AML continues to strengthen its partnerships and develop resources. Future efforts include creating an online continuing education course for healthcare providers and expanding health literacy initiatives for immigrant, refugee, and Alaska Native communities. These ongoing projects highlight AML's commitment to improving health literacy, combating misinformation, and enhancing access to health information across the vast and geographically challenging state of Alaska.

## PP4. Dispensing research skills: enhancing PharmD students' evidence-based practice

Caitlin Carter, Sarah Fallis, Nardine Nakhla
University of Waterloo

**Introduction:** Developing critical literature review skills is essential for pharmacy students to provide evidence-based patient care. In winter 2024, the course coordinator of the Advanced Patient Self-Care elective invited the librarian to co-teach a literature review tutorial with the course teaching assistant. As part of this elective, students (in small groups) were required to conduct a literature review, as well as use their results to create a clinical tool for pharmacists. **Methods:** Twenty third-year PharmD students enrolled in the elective participated in the optional one-hour tutorial, developed and co-taught by the librarian and teaching assistant. The tutorial covered an overview of literature review methods, such as research question, search strategy and eligibility criteria development, and critical appraisal. After the tutorial, the librarian and teaching assistant provided 1-on-1 support to each small group and were available for continued support leading up to the assignment deadlines. Effectiveness was assessed through student questions and feedback, as well as assignment outcomes. **Outcomes:** Students demonstrated increased confidence in using PubMed and constructing search strategies. Most reported finding the librarian's involvement effective and felt more comfortable conducting literature reviews. Three resulting clinical tools were selected for publication in a pharmacy practice magazine. Overall, student performance on assignments improved. **Discussion:** This interdisciplinary collaboration proved highly effective in enhancing students' literature review skills and learning experience. This successful model has become a regular feature of the elective and could serve as a template for other librarian-faculty partnerships in instruction across various disciplines.

## PP5. Distributed librarianship: supporting UBC's rehabilitation students across BC

Rachael Bradshaw[1], Aubrey Geyer[1], Zahra Premji[2]
[1]University of British Columbia, [2]University of Victoria

**Background:** UBC is the only academic institution in British Columbia that provides accredited master's degrees for rehabilitation professionals in Physical Therapy, Occupational Therapy, and Audiology and Speech Sciences. British Columbia is struggling with a shortage of rehabilitation professionals, leading to the expansion of UBC's rehabilitation programs to satellite sites in Surrey, Prince George, and Victoria (Health Sciences Association of British Columbia, 2021). **Case Presentation:** UBC Library has adopted a distributed model of librarianship to match this distributed education model. The model was developed by librarians who support UBC's distributed Undergraduate Medicine program, but it has only been adopted by rehabilitation sciences within the last five years. In practice, our model consists of one coordinating librarian who supports the Vancouver cohort of each program and oversees the coordination of teaching, collections, and resource development for the entire program. Each distributed site has its own on-site librarian who supports their cohort as one part of their multi-faceted roles. **Conclusions:** This presentation will describe our model of library support for UBC's continually developing rehabilitation programs. We will outline the benefits of the model, including improving the learning experience for students through onsite teaching, reducing the burden of keeping up with program expansions, and bringing multiple perspectives into our work. We will also discuss challenges that we have faced, such as coordination with instructors at multiple sites and technical issues with hybrid teaching. Though this model is not without its challenges, it is nevertheless a novel way to ensure that all students in these distributed programs have equitable access to library support.

## References

1. Health Sciences Association of British Columbia. (2021, October). 'We're chronically understaffed': A report on public rehabilitative healthcare in BC. https://hsabc.org/sites/default/files/2022-10/hsa-chronically-understaffed-exec-summary-for-web.pdf.

## PP6. Effect of citation numbers and team members on the likelihood of and time needed to complete screening for systematic and scoping reviews

Emma Barrett-Catton, Emily Jones, Rebecca Carlson
University of North Carolina at Chapel Hill

**Objective:** To identify the effect that the total number of citations and team members has on the likelihood of completion and time needed for screening. **Methods:** We obtained institutional review data of a large research university from Covidence. Data included review name, type, area, date created and last active, number of collaborators, presence of librarian collaboration, and the number of citations imported, screened, and removed at each step. Data were cleaned to remove items that were not true reviews and were analyzed using SPSS linear regression and independent sample Mann-Whitney U tests. Outcomes included the effect of number of total citations, number of citations per collaborator, and librarian collaboration on the percentage screened and time needed to complete title/abstract and full-text screening. **Results:** The fewer citations and the fewer citations per collaborator, the more likely the team is to complete title/abstract and full-text screening, and the faster they will finish the screening process. This relationship was stronger for number of citations per collaborator than number of citations alone. There was no significant difference between the percentage screened in title/abstract for reviews with versus without librarian collaboration. However, reviews without librarian collaboration had significantly higher median percentage of full texts screened. **Conclusions:** This study allows librarians to provide more informed guidance to teams on elements that may increase the likelihood of screening completion for systematic and scoping reviews. It emphasizes the importance of narrowing the scope of a review or increasing the size of the team to make screening completion more achievable.

## PP7. Finding the S (studies) before the R (reports) in SRs of intervention effects

Zahra Premji[1], Chris Cooper[2], Christine Worsley[3], Eve Tomlinson[2], Sarah Dawson[2]. Emma Prentice[3]
[1]University of Victoria, [2]University of Bristol, [3]Tolley Ltd.

**Objective:** To describe a new process model of study identification specifically for randomized studies in systematic reviews of intervention effect. **Methods:** Identification of studies is a critical step in the conduct of systematic reviews of effectiveness. The prevailing approach to study identification in systematic reviews, referred to as 'The Conventional Approach,' (Cooper et al., 2018) prioritizes bibliographic database searching as the primary method of study identification, followed by searches of grey literature including registers and conferences, and supplementary search methods. Studies and study reports identified by all of these methods are then pooled for study selection. A new process model is proposed which separates the search for studies from the search for study reports, into distinct phases. We distinguish here between studies and study reports, the former being the focus of the first phase in this process model. **Results:** The proposed three phase process model will be described and illustrated. The implications of early study identification in phase one, on the subsequent bibliographic database search in phase two, will be highlighted. **Conclusions:** This new process model is an alternate to The Conventional Approach of study identification for use in complicated systematic reviews of intervention effectiveness.

## References

1. Cooper C, Booth A, Varley-Campbell J, Britten N, Garside R. Defining the process to literature searching in systematic reviews: a literature review of guidance and supporting studies. BMC Medical Research Methodology 2018;18(1).

## PP8. How on EARTH do you assess risk of bias in information retrieval studies? We didn't know either, so we drafted a tool

Chris Cooper[1], Eve Tomlinson[1], Zahra Premji[2], Anna Brown[3], Rachel Court[3]
[1]University of Bristol, [2]University of Victoria, [3]University of Birmingham

**Background:** We are undertaking a Cochrane review evaluating the effect of supplementary search methods compared to bibliographic searching to identify randomised studies. Cochrane requires an appraisal of the risk of bias of studies included in their systematic reviews. The standard tool recommended by Cochrane (RoB 2) covers interventions evaluated in randomised studies. This 'type' of study does not align with the information retrieval studies we anticipate finding (comparative case studies), so we designed our own risk of bias tool specific to information retrieval (IR) studies. **Methods:** We undertook a review of the following risk of bias tools identified via the Latitudes website.(1) The aim of the review was to determine the applicability of these tools to IR studies:

- RoB 2;

- EPOC - suggested risk of bias criteria for EPOC reviews [proposed criteria for RCTs, non-RCT, and controlled before-after]
- ROBIS
- Newcastle-Ottawa Quality Assessment Scale Case Control Studies
- Scale for the Assessment of Narrative Review Articles (SANRA)

This review was then supplemented by a methodological review by Tomlinson and colleagues who evaluated common challenges and suggestions for risk of bias tool development.(2) **Results:** Our proposed tool has three domains: review of study protocol, the comparison, and outcomes. We provide guidance on how to judge risk of bias, adopting the signaling questions from the ROBIS tool. **Conclusions:** This is the first tool to appraise risk of bias in IR studies. We will report the tool in full for the first time, providing a worked example of the tool and findings.

## References

1. LATITUDES Network. LATITUDES Network. 2017. URL: https://www.latitudes-network.org/about/ (Accessed September 2024).
2. Tomlinson E, Cooper C, Davenport C, Rutjes AWS, Leeflang M, Mallett S, et al. Common challenges and suggestions for risk of bias tool development: a systematic review of methodological studies. Journal of Clinical Epidemiology 2024;171111370.

## PP9. Library of Search Strategy Resources (LSSR): bringing together search resources for literature searching

Mark Mueller
Saskatchewan Health Authority

The Library of Search Strategies Resources (LSSR) website is an open access resource for anyone sourcing and developing search strategies for health science related topics. While there are many sites and repositories that provide access to filters and hedges on the web (i.e. ISSG) to help with the duplication of efforts and topical overlaps, sometimes they are difficult to find and/or not immediately accessible to the public. With this in mind, the purpose of the LSSR is to provide searchers access to a comprehensive and centralized resource of dispersed resources for search filter development. The "Collections" page lists databases and collections (i.e. ISSG) where users can find search filters that they can adapt and integrate into their needs. The LSSR also contains links to free online tools, quality assurance resources, learning resources that users can use to support search strategy development. The LSSR is a resource designed to assist searchers at any stage of the search filter development process. The LSSR was developed by an international team of librarians passionate about searching. With representation from Canada, United States, Western Europe, and other parts of the world, our aim is to create a resource for a global audience. Future projects for the LSSR include building strategic partnerships professional organizations; monitoring emerging technologies; and creating communities of practice to assist with the development of new and innovative content.

## PP10. Obtaining grants in health sciences librarianship: advice, approach, and strategies for librarian researchers

**Background:** Grant writing in health sciences librarianship is intricate and often daunting, particularly for first-time applicants. Despite its critical importance in advancing library resources and services, limited literature exists on this domain's specific challenges and strategies. This project reflects on personal experiences, lessons learned, and evidence-informed recommendations for navigating health sciences librarianship's grant writing and funding landscape. **Description:** The grant application process involves multiple stages, from conceptualizing research ideas to responding to critiques following a rejection. Rejection is common, even among seasoned researchers, but it need not signal the end of a grant's journey. Instead, it presents an opportunity to refine the proposal, address reviewers' feedback, and resubmit a stronger application. Key steps in this iterative process include consulting co-investigators for expert insights, refining hypotheses to align with funding priorities, and drafting concise introductory statements communicating the project's significance and objectives. **Program Conclusion:** Our recommendations underscore the importance of understanding and leveraging the peer review process to your advantage. By embracing rejection as a natural step toward eventual success, librarian researchers can transform challenges into opportunities. Persistence, strategic revisions, and adaptability are essential to securing funding and advancing novel ideas in health sciences librarianship. This project aims to inspire and empower librarians to approach grant writing with resilience and strategic intent, ultimately enhancing their ability to secure funding for impactful library initiatives.

## PP11. Still a filtering failure? Automated indexing using MTIX versus MTIA and its impact on human study filtering for knowledge synthesis

Nicole Askin, Tyler Ostapyk, Carla Epp
University of Manitoba

**Introduction:** The search filter 'exp animals/ not humans.sh' is a well-established method in knowledge synthesis used to exclude non-human studies in Ovid Medline. We previously reported on the impact of the Medical Text Indexer-Auto (MTIA) algorithm for automated assignment of MeSH terms on the utility of this filter for knowledge synthesis projects. We sought to update our reporting to account for the 2024 implementation of the new Medical Text Indexer-NeXt Generation (MTIX) algorithm, which uses a machine-learning model for MeSH term assignment. **Methods:** As in the previous study, we conducted a search in Ovid Medline using the Cochrane Highly Sensitive Search Strategy. We isolated the results indexed by the automated method and specifically excluded by the non-human-studies filter in the timeframe since MTIX was implemented. We screened these results using Covidence to identify human studies. **Results:** The sample demonstrated a significant improvement over our assessment of MTIA: only 1% (25/2285) of studies screened were inappropriately excluded human studies - compared to 4.2% in the MTIA assessment - and none of these were in a clinical context. Records describing both animal and human studies continue to be a common source of inappropriate exclusion. **Discussion:** Our findings suggest that the filter is much less likely to inappropriately exclude human studies indexed by MTIX (records indexed beginning April 2024) than MTIA (studies indexed between 2019 and April 2024). However, we still recommend caution with the use of the human studies filter, especially for records indexed between 2019-2024.

JOURNAL INFORMATION
==============================
NLM Title Abbreviation: J Can Health Libr Assoc
Journal ID: J Can Health Libr Assoc
NLM Journal ID: 101229936
Journal ID: JCHLA

The Journal of the Canadian Health Libraries Association

EISSN: 1708-6892
Publisher: Canadian Health Libraries Association / Association des bibliotèques de la santé du Canada

ARTICLE INFORMATION
==============================
Subjects: PP = Poster Presentation

PP1. A fork in the road: machine learning classifier or methodological search filter to identify systematic reviews?

Severn Melissa
Epworth Alissa
Adams Romney
DistillerSR

JOURNAL INFORMATION
==============================
NLM Title Abbreviation: J Can Health Libr Assoc
Journal ID: J Can Health Libr Assoc
NLM Journal ID: 101229936
Journal ID: JCHLA

The Journal of the Canadian Health Libraries Association

EISSN: 1708-6892
Publisher: Canadian Health Libraries Association / Association des bibliotèques de la santé du Canada

ARTICLE INFORMATION
==============================
Subjects: PP = Poster Presentation

PP2. A review of library services and supports for the University of Prince Edward Island's (UPEI) growing One Health programs

McCaffrey Keri
Mears Kim
University of Prince Edward Island

JOURNAL INFORMATION
==============================
NLM Title Abbreviation: J Can Health Libr Assoc
Journal ID: J Can Health Libr Assoc
NLM Journal ID: 101229936
Journal ID: JCHLA

The Journal of the Canadian Health Libraries Association

EISSN: 1708-6892
Publisher: Canadian Health Libraries Association / Association des bibliotèques de la santé du Canada

ARTICLE INFORMATION
==============================
Subjects: PP = Poster Presentation

PP3. Building a healthier Alaska: connecting Alaskans to essential resources through the Alaska Medical Library

Bjartmarsdottir Anna
McKay Jennifer
University of Alaska

JOURNAL INFORMATION
==============================
NLM Title Abbreviation: J Can Health Libr Assoc
Journal ID: J Can Health Libr Assoc
NLM Journal ID: 101229936
Journal ID: JCHLA

The Journal of the Canadian Health Libraries Association

EISSN: 1708-6892
Publisher: Canadian Health Libraries Association / Association des bibliotèques de la santé du Canada

ARTICLE INFORMATION
==============================
Subjects: PP = Poster Presentation

PP4. Dispensing research skills: enhancing PharmD students' evidence-based practice

Carter Caitlin
Fallis Sarah
Nakhla Nardine
University of Waterloo

JOURNAL INFORMATION
==============================
NLM Title Abbreviation: J Can Health Libr Assoc
Journal ID: J Can Health Libr Assoc
NLM Journal ID: 101229936
Journal ID: JCHLA

The Journal of the Canadian Health Libraries Association

EISSN: 1708-6892
Publisher: Canadian Health Libraries Association / Association des bibliotèques de la santé du Canada

ARTICLE INFORMATION
==============================
Subjects: PP = Poster Presentation

PP5. Distributed librarianship: supporting UBC's rehabilitation students across BC

Bradshaw Rachael 1
Geyer Aubrey 1
Premji Zahra 2
1 University of British Columbia,
2 University of Victoria

JOURNAL INFORMATION
==============================
NLM Title Abbreviation: J Can Health Libr Assoc
Journal ID: J Can Health Libr Assoc
NLM Journal ID: 101229936
Journal ID: JCHLA

The Journal of the Canadian Health Libraries Association

EISSN: 1708-6892
Publisher: Canadian Health Libraries Association / Association des bibliotèques de la santé du Canada

ARTICLE INFORMATION
==============================
Subjects: PP = Poster Presentation

PP6. Effect of citation numbers and team members on the likelihood of and time needed to complete screening for systematic and scoping reviews

Barrett-Catton Emma
Jones Emily
Carlson Rebecca
University of North Carolina at Chapel Hill

JOURNAL INFORMATION
==============================
NLM Title Abbreviation: J Can Health Libr Assoc
Journal ID: J Can Health Libr Assoc
NLM Journal ID: 101229936
Journal ID: JCHLA

The Journal of the Canadian Health Libraries Association

EISSN: 1708-6892
Publisher: Canadian Health Libraries Association / Association des bibliotèques de la santé du Canada

ARTICLE INFORMATION
==============================
Subjects: PP = Poster Presentation

PP7. Finding the S (studies) before the R (reports) in SRs of intervention effects

Premji Zahra 1
Cooper Chris 2
Worsley Christine 3
Tomlinson Eve 2
Dawson Sarah 2
Prentice Emma 3
1 University of Victoria,
2 University of Bristol,
3 Tolley Ltd.

JOURNAL INFORMATION
==============================
NLM Title Abbreviation: J Can Health Libr Assoc
Journal ID: J Can Health Libr Assoc
NLM Journal ID: 101229936
Journal ID: JCHLA

The Journal of the Canadian Health Libraries Association

EISSN: 1708-6892
Publisher: Canadian Health Libraries Association / Association des bibliotèques de la santé du Canada

ARTICLE INFORMATION
==============================
Subjects: PP = Poster Presentation

PP8. How on EARTH do you assess risk of bias in information retrieval studies? We didn't know either, so we drafted a tool

Cooper Chris 1
Tomlinson Eve 1
Premji Zahra 2
Brown Anna 3
Court Rachel 3
1 University of Bristol,
2 University of Victoria,
3 University of Birmingham

JOURNAL INFORMATION
==============================
NLM Title Abbreviation: J Can Health Libr Assoc
Journal ID: J Can Health Libr Assoc
NLM Journal ID: 101229936
Journal ID: JCHLA

The Journal of the Canadian Health Libraries Association

EISSN: 1708-6892
Publisher: Canadian Health Libraries Association / Association des bibliotèques de la santé du Canada

ARTICLE INFORMATION
==============================
Subjects: PP = Poster Presentation

PP9. Library of Search Strategy Resources (LSSR): bringing together search resources for literature searching

Mueller Mark
Saskatchewan Health Authority

JOURNAL INFORMATION
==============================
NLM Title Abbreviation: J Can Health Libr Assoc
Journal ID: J Can Health Libr Assoc
NLM Journal ID: 101229936
Journal ID: JCHLA

The Journal of the Canadian Health Libraries Association

EISSN: 1708-6892
Publisher: Canadian Health Libraries Association / Association des bibliotèques de la santé du Canada

ARTICLE INFORMATION
==============================
Subjects: PP = Poster Presentation

PP10. Obtaining grants in health sciences librarianship: advice, approach, and strategies for librarian researchers

Ross-White Amanda 1
Iansavitchene Alla 2
1 Queen's University,
2 London Health Sciences Centre

JOURNAL INFORMATION
==============================
NLM Title Abbreviation: J Can Health Libr Assoc
Journal ID: J Can Health Libr Assoc
NLM Journal ID: 101229936
Journal ID: JCHLA

The Journal of the Canadian Health Libraries Association

EISSN: 1708-6892
Publisher: Canadian Health Libraries Association / Association des bibliotèques de la santé du Canada

ARTICLE INFORMATION
==============================
Subjects: PP = Poster Presentation

PP11. Still a filtering failure? Automated indexing using MTIX versus MTIA and its impact on human study filtering for knowledge synthesis

Askin Nicole
Ostapyk Tyler
Epp Carla
University of Manitoba

